# Correction: Ambulatory management of pre- and extensively drug resistant tuberculosis patients with imipenem delivered through port-a-cath: A mixed methods study on treatment outcomes and challenges

**DOI:** 10.1371/journal.pone.0299477

**Published:** 2024-02-21

**Authors:** Vijay Vinayak Chavan, Alpa Dalal, Sharath Nagaraja, Pruthu Thekkur, Homa Mansoor, Augusto Meneguim, Roma Paryani, Pramila Singh, Stobdan Kalon, Mrinalini Das, Gabriella Ferlazzo, Petros Isaakidis

There are errors in the Funding and Competing Interests statements. The correct statements are:

**Funding:** Médecins Sans Frontières (MSF) provided support in the form of salaries for VVC, HM, AM, RP, PS, SK, MD, GF, and PI. The specific roles of these authors are articulated in the ‘author contributions’ section. MSF programmatic funding covered all other costs of the study. The funder was involved in study design, data collection and analysis, decision to publish, and preparation of the manuscript.

**Competing Interests:** The authors have read the journal’s policy and have the following competing interests to declare: Médecins Sans Frontières (MSF) provided support in the form of salaries for VVC, HM, AM, RP, PS, SK, MD, GF, and PI. This does not alter our adherence to *PLOS ONE* policies on sharing data and materials. There are no patents, products in development, or marketed products associated with this research to declare.
