# Supplementary material for: Ambulatory management of pre- and extensively drug resistant tuberculosis patients with imipenem delivered through port-a-cath: A mixed methods study on treatment outcomes and challenges
Source: PLoS One. 2020 Jun 16;15(6):e0234651. doi: 10.1371/journal.pone.0234651 (PMC7297304; doi:10.1371/journal.pone.0234651)
Supplement: Attachment — Submitted filename: Response to reviewers (2).doc [file pone.0234651.s001.doc]

January 23^rd^, 2020 - Responses to points brought up by the reviewer

**Title:** Ambulatory management of pre and extensively drug resistant tuberculosis patients with Imipenem delivered through port-a-cath : A mixed methods study on treatment outcomes and challenges.

**Journal: Plos One**

**Submission ID: PONE-D-19-25948**

Dear Editor,

We thank you and the reviewer for reviewing our paper. We are pleased that there is interest in the topic and are grateful for the constructive comments that could make the manuscript suitable for publication.

We have addressed you and the reviewers’ comments point-by-point below and have made corresponding changes in the manuscript under ‘tracked changes’ to indicate the revised portions of the manuscript.

Sincerely,

Vijay V Chavan

Corresponding author

**Comments received by Editor:**

***Comment 1:*** Please remove all the data of the qualitative study, to be published in a different paper, and stick only to the quantitative data, as explained by the reviewer in this case.

***Response:*** Thank you very much for your suggestion. We conceptualized this study as a mixed method study with quantitative and qualitative component. We understand the use of Port-a-cath device for Imipenem infusion was a novel technique and even the quantitative description of the treatment outcomes with such techniques has a merit for scientific publication. However, the difficulties with the long term injectable treatment and many challenges in delivering imipenem through this novel technique needs to be highlighted for replication and improving the package further. Thus, the study was aimed to holistically bring out the outcomes and challenges with Imipenem treatment for patients with drug resistant tuberculosis.

Previous studies have reported on the treatment outcomes of Imipenem use in drug resistant tuberculosis patients treated with hospitalization for the entire duration of Imipenem infusion. Our study is the first to report on the possibility to deliver Imipenem on ambulatory basis for drug resistant TB patients when all oral regimens could not be given. The treatment outcomes of patients on treated on ambulatory basis is encouraging. Hence, not reporting on challenges in delivering the treatment on ambulatory basis could provide a wrong impression and expectation from the treatment package. Thus, our team feels very strongly that we need to present our quantitative results with the qualitative part on challenges to enable comprehensive understanding of the treatment package. The research protocol including both the quantitative and qualitative components received approval from the ethics review boards, and we would like to present findings of both the components in the single manuscript.

**Comments received by Reviewer 1:**

**General:**

***Comment 1:*** It was a pleasure to review your manuscript. You have performed a great job in the field and high quality research. My main concerns regarding the manuscript, in general, is that you are trying to present in the same manuscript two what is seems to me, two different studies using mixed methods study (one quali and other quantitative). It lengthens the text and causes it to be in some unstructured and confusing parts. I would suggest splitting both parts and try to publish qualitative and quantitative parts separately.

***Response:*** Thank you very much for your suggestion. We conceptualized this study as a mixed method study with quantitative and qualitative component. We understand the use of Port-a-cath device for Imipenem infusion was a novel technique and even the quantitative description of the treatment outcomes with such techniques has a merit for scientific publication. However, the difficulties with the long term injectable treatment and many challenges in delivering imipenem through this novel technique needs to be highlighted for replication and improving the package further. Thus, the study was aimed to holistically bring out the outcomes and challenges with Imipenem treatment for patients with drug resistant tuberculosis.

Previous studies have reported on the treatment outcomes of Imipenem use in drug resistant tuberculosis patients treated with hospitalization for the entire duration of Imipenem infusion. Our study is the first to report on the possibility to deliver Imipenem on ambulatory basis for drug resistant TB patients when all oral regimens could not be given. The treatment outcomes of patients on treated on ambulatory basis is encouraging. Hence, not reporting on challenges in delivering the treatment on ambulatory basis could provide a wrong impression and expectation from the treatment package. Thus, our team feels very strongly that we need to present our quantitative results with the qualitative part on challenges to enable comprehensive understanding of the treatment package. The research protocol including both the quantitative and qualitative components received approval from the ethics review boards, and we would like to present findings of both the components in the single manuscript.

***Comment 2:*** Other concerns are that the introduction and discussion sections need to state more clearly the priority of implementation of new guidelines of M/XDR TB management launched by WHO using new drugs and regimens leaving injectables and iv treatment use as a secondary or tertiary alternatives (E.g.: new regimens with Linezolid, Bedaquiline, Delamanid, Pretomanid will probably could bring better treatment outcomes with less adverse events if possible to use.

***Response:*** Thank you for the comment. We have stated the WHO recommendations on the newer drugs in the line number 105-114 of the introduction. We have also given reference for WHO drug basket for management of drug resistant TB.

***Comment 3:*** Introduction section is too long and discussion too short and needs more work and order. Some parts of the analysis should be reworked and some references need to be up dated.

***Response:*** Thank you for this suggestion. We have looked into it carefully and incorporated the suggestions in the revised manuscript. Specific changes made are highlighted in the specific comments.

***Comment 4:*** Please, also note that even though patients and probably data were collected in a prospective manner, this is not a cohort study as it is reflected in the data analysis. If you would like to consider a cohort study, I would suggest changing analysis trying to incorporate the variable time and calculate Hazard or Risk Ratios.

***Response:*** Thank you for highlighting this deficiency. We acknowledge that this was not a pure ‘cohort study’ as we did not intend to test any hypothesis. We have changed the study design as a ‘longitudinal descriptive study’.

***Comment 5:*** Saying that I would like to underline that operational research by NGOS such is MSF in high burden M/XDR TB countries is essential as it is well demonstrated in the study. Even is not the aim of the study, it is also a cost-effective strategy treating and following in a very good way a high number of patients. After addressing the main comments and the following minor issues, the manuscript could be ready to be published.

***Response:*** Thank you for appreciating the work.

**Specific comments:**

***Comment 1:*** Title: according with my general comment, I’d suggest to delete the second part of the title “…a mixed methods study on treatment outcomes and challenges”.

***Response:*** Thank you for the suggestion. As we strongly feel to include the qualitative party in the manuscript, we wish to retain the title as it is.

***Comment 2:*** Keywords: should be MeSH Terms in alphabetic order.

***Response:*** Thank you for the suggestion. We have now provided appropriate MeSH Term as Keywords and have arranged these in alphabetical order. However, the ‘Operational research’ and ‘SORT IT’ doesn’t have a specific MeSH Terms and are retained as the last two ‘Keywords’. Changes made in line number 91 and 92.

***Comment 3:*** Abstract: Please, review this carefully considering the general comments.

***Response:*** Thank you for the comment. We have not made major changes as we have retained the manuscript as a mixed-methods study. However, we have revisited the quantitative study design and have changed it to ‘longitudinal descriptive study’. Changes made in line number 72-73.

**Introduction:**

**Comment 4:** Well written and structured but too long. Try to shorten to 1 page. Up-date data and references number 1, 4, 5.

***Response:*** Thank you for the comment. We have changed the reference-1 with the latest reference. However, we feel the reviews in reference 4 (published in 2012) and 5 (published in 2008) are important references for the corresponding line in the manuscript. These were the two manuscripts which questioned strongly the standardized regimen for management of drug resistant TB. Hence, we have retained these references.

***Comment 5:*** Line 102: add “…cases due to safety of second line drugs and to…”

***Response:*** Thank you for the suggestion. Suggested change has been made.

***Comment 6:*** Lines 110-115: see and comment new WHO recommendations. This paragraph is not refereeing to the new recommendations.

***Response:*** Thank you for the suggestion. At the time of drafting this paper (September 2019), the new guidelines was not present as it was made available in November 2019. However, we have revisited this paragraph and have provided the new reference.

***Comment 7:*** Redefine the objective: delete objective number 2 and add in objective number 2: “…and compare new drugs +imipenem with other no new drugs with imipenem…”

Author: Thank you for the suggestion. We have not deleted the objective-2 as we wish to retain the qualitative component in the manuscript. Also, we have not included the suggested objective as the study was not designed to compare the treatment outcomes between two groups. All the patients included in the study received atleast one new drug in their regimen with Imipenem, thus the suggested objective/analysis will not be possible for the study. The patients were divided into two groups: One with Imipenem and both new drugs (IBDLC) while other with Imipenem and either Bedaquiline or Delamanid. This is also described in Table No. 5 of the manuscript.

**Methods:**

***Comment 8:*** Well-structured but seven pages are too much. Please, delete the qualitative part and try to shorten this section.

***Response:*** Thank you for the suggestion. However, as we wish to include the qualitative part, we have retained the structure as it is.

***Comment 9:*** It seems that this is an observational analytical cross-sectional study instead of cohort study.

***Response:*** Thank you for the suggestion. This was an observational study with longitudinal follow-up of patients from treatment initiation to outcome. Thus, we have revisited the study design and have changed it to ‘longitudinal descriptive study’.

***Comment 10:*** Setting: Is MSF covering all territory? Try to define the population/area of coverage. Are patients studied representative of the total/general population? Are cases representatives of the general population? This is a key issue for the external validity of the study. Please discuss.

***Response:*** MSF independent clinic receives referrals for treatment from different areas of Mumbai city. Thus we believe patients are representative of the patients with drugs resistant tuberculosis in Mumbai and have severe drug resistant TB pattern.

***Comment 11:*** Figure number 1 has a very bad quality. Impossible to read.

***Response:*** Thank you for the suggestion. We have revised the Figure-1.

***Comment 12:*** Follow up and adverse events part: are you describing the standard of care? If so, you can refer to already published guidelines.

***Response:*** Thank you for the suggestion. We have described the follow-up and adverse events as there is no published literature on the standard of care from the study setting. Also, as this is newer technique and regimen there are no global recommendations for follow-up. Thus, we feel the description is essential as the results have to be interpreted in line with the protocol followed in the study setting.

***Comment 13:*** What definitions are you using for treatment outcomes definition? Please, add a reference to support these definitions. If the definitions are the standard accepted, refer to the readers to the published definitions should be enough.

***Response:*** Thank you for the comment. The outcomes were adapted from the technical and operational guidelines for management of TB in India. However, we wish to retain the Table-1 as few of the outcomes were improvised based on the requirement of regimen in use. Changes made in line number 244-245

***Comment 14:*** Study population: same comment above; is all population of Bombay or any defined district?

***Response:*** Thank you for the comment. As mentioned earlier, the patients are referred to MSF clinic from anywhere in Mumbai. Thus, the DR-TB patients are not restricted to any districts within Mumbai.

***Comment 15***: Source and data analysis: Include only the analyzed variables and in the same order that are stated in the results section, ideally: demographic, social, risk factors, diagnostic, treatment…

***Response:*** Thank you for the comment. We have revisited this section and have detailed it according the results section. Changes made in line number 290 to 297.

***Comment 16:*** Add the comparative analysis performed new drugs + imipenem with other no new drugs with imipenem. Give the 95% CI.

***Response:*** Thank you for the suggestion. However, as mentioned earlier, we did not intend to compare the treatment outcomes between two groups.

***Comment 17:*** Delete the part about time to culture conversion. I cannot see any results in this regard.

***Response:*** Thank you for the comment. We have detailed on the culture conversion in results section in line number 356 to 358.

***Comment 18:*** It is better to use the median and interquartile range if the continuous variables do not follow normal distribution.

***Response:*** Thank you for the comment. We have used median and interquartile for summarizing continuous variables except for age, which followed normal distribution.

**Results:**

***Comment 19:*** Please, add relapse definition in table 2.

***Response:*** Thank you for the comment. We have added a definition of relapse in the footnote of Table-2.

***Comment 20:*** Table 4: adding two more columns with the two regimens (IBDLC and other) would give very good information.

***Response:*** Thank you for the suggestion. However, just disaggregating with IBDLC and other may not be able to relate the events to any specific drug. As describe earlier, the ‘other’ also included newer drugs in different combination.

***Comment 21:*** Table 5 very important: add a column with the p-values.

***Response:*** Thank you for the comment. As described earlier, the intention of the study was not to compare the treatment outcomes between these groups rather to describe it.

**Discussion:**

***Comment 22:*** Try to put a little order following the results section.

***Response***: Thank you for the suggestion. We have structured our discussion under these broad headings- Key Findings (1 paragraph), Comparision of key findings with other literature (3 paragraphs), strengths of the study (1 paragraph), limitations of the study (1 paragraph) and implications plus recommendations (5 paragraphs). The key findings are in line with the flow of the results.

***Comment 23:*** When you talk about imipenem, are you referring always to the combination with amoxiclav? What doses?

***Response:*** Thank you for this query. Imipenem is always prescribed with amoxicillin-clavulanate (500mg + 125mg) twice daily. We have detailed about it in line number 209-210.

***Comment 24:*** Regarding the proportion of culture conversion (26/36 patient’s culture positive) at imipenem initiation, I think is difficult to attribute it to only imipenem action. Would it be also possible to the activity of other more active anti-TB drugs (BM, Bq, Lz,..)? Please discuss.

***Response:*** Regarding sputum conversion and also treatment outcomes, we definitely agree that these cannot be attributed to Imipenem alone. Drug Resistant Tuberculosis treatment has many drugs in the regimen and therefore favorable outcomes are attributed to regimens and not to a single drug; Imipenem in our study. Injectable drug Imipenem was added to the regimen only when an effective drug regimen was not feasible with available drugs considering the sensitivity, prior drug exposure and adverse events to any drug in the regimen. Thus, we have added this as one of our limitation now. Changes made in line number 524-527

***Comment 25:*** Lines 470-471 and Table 5: when you compare both regimens IBDLC and other imipenem regimen, was the duration the same for both? Median of duration for both regimens? Any differences?

***Response:*** Thank you for the comment. As described in the setting, the decision to stop imipenem was based on the microbiological and clinical improvement. Thus, we did not attempt to calculate the duration of treatment. When checked now, there was no difference between the two.

***Comment 26:*** Lines 478-479: QTc prolongation definition not here. Line 482: severe cardiac event definition?

***Response:*** QT interval was calculated by Fredricia method and called prolonged when the interval was more than 500 milliseconds. The definition of severe cardiac event: Severe cardiac event with newer drugs like BDQ and DLM with High dose Moxifloxacin and Clofazimine (all QT interval Prolonging drugs) was mostly arrhythmias. The definitions are added in line number 207 to 209.

***Comment 27:*** Lot of new and good ideas discussed. Left some of them as a recommendations (eg: lines 516, 521, etc.

***Response:*** Thank you for the appreciation. As mentioned earlier, we have made all the suggestion for NTPs under the implications and recommendation section of our discussion.

***Comment 28:*** Comment and compare the favorable outcome with those already published (eg: Nix TB trial).

***Response:*** Thank you for the comment. We had not commented on the Nix-TB trial as the results were not updated. Now we have mentioned it in our manuscript and have compared it with our results, Changes made in line number 486-488.

***Comment 29:*** Please, try to redefine the conclusion according to the aim of the study. From my perspective, 65% of favorable outcome is not as promising as you state in the conclusion section.

***Response:*** According to the Annual India TB report, XDR TB patients initiated during 3Q15 to 2Q16, success rate was reported to be 27% with 42% death and 14% loss to follow up. Our 65% favorable outcome when compared to the national outcome does prove to be promising. However, now we have given a justification for our interpretation by providing the comparison with outcomes that reported in annual TB report. Changes made in line number 526-529.

***Comment 30:*** Complete the manuscript with a few of important recommendations deducted from the study would be desirable.

***Response:*** Thank you for the comment. All the study implications mentioned in the discussion section are followed with relevant recommendations. Thus, we don’t want to repeat it again as a separate entity.
