# Supplementary material for: Ambulatory management of pre- and extensively drug resistant tuberculosis patients with imipenem delivered through port-a-cath: A mixed methods study on treatment outcomes and challenges
Source: PLoS One. 2020 Jun 16;15(6):e0234651. doi: 10.1371/journal.pone.0234651 (PMC7297304; doi:10.1371/journal.pone.0234651)
Supplement: Attachment — Submitted filename: Response to reviewer R2.doc [file pone.0234651.s002.doc]

May 6^th^, 2020 - Responses to points brought up by the reviewer_R2

**Title:** Ambulatory management of pre and extensively drug resistant tuberculosis patients with Imipenem delivered through port-a-cath: A mixed methods study on treatment outcomes and challenges.

**Journal: Plos One**

**Submission ID: PONE-D-19-25948R1**

Dear Editor,

We thank the reviewer for reviewing our paper. We have addressed the reviewers’ comments point-by-point below and have made corresponding changes in the manuscript under ‘tracked changes’ to indicate the revised portions of the manuscript.

Sincerely,

Vijay V Chavan

Corresponding author

**Response to Reviewer 1:**

***General:***

***Point 1:*** This is a well-researched manuscript by using mixed method design and multiple data sources on one of a major public health problem. However, it requires some clarifications and to make more comprehensive as well as be consistent throughout the document.

***Response 1:*** We are grateful to the reviewer for the constructive comments and suggestions.

***Specific Comments:***

***Abstract:***

***Point 2:*** Abstract method is not complete, only mentioned design, better to include study population, sampling, data collection and analysis in short.

***Response 2:*** We have revised the methods section in abstract of the manuscript.

***Point 3:*** Line 73 “… qualitative (descriptive study…)”. Is it explorative or descriptive?

***Response 3:*** We believe our study design is descriptive as both the components were carried out concurrently (convergent) but the results of one component did not influence the queries/methodology of other component.

***Point 4:*** Line 76 “.51 (72.9%) had XDR-TB” --- start any sentence with words not with number, Fifty one …

***Response 4:*** We agree with the reviewer and the number has been changed to word in the abstract.

***Introduction***

***Point 5:*** Line 127 “ … its use in management of DR-TB is not documented.” Not documented where? In the study area or at all (Globally)? Need clarification. Need to add more similar or related studies conducted elsewhere.

***Response 5:*** To our knowledge, the use of port-a-cath in management of MDR-TB has not been documented globally. There are studies reporting on Imipenem administration for treatment of patients with MDR-TB, but none have used port-a-cath as a medium of administration.

***Methods:***

***Point 6:*** Under general Setting, lines 155 – 157, mention how many public health facilities, NGOs/private clinics are providing the free care to TB patients. Also how many of these facilities are providing ambulatory management of pre and extensively drug resistant TB patients?

***Response 6:*** In India, free of charge TB treatment is provided in all public health facilities under national TB programme. To share the load of overwhelming public facilities, select NGOs (focused in TB) provide free TB care to patients. Though in the country a significant share of patients received treatment in private sector, it will be difficult to mention the number of facilities providing the treatment.

***Point 7:*** Study population for qualitative, lines 235 and 237, you employed purposive sampling technique for both health care providers and for patients, please specify the type of purposive sampling used for each.

***Response 7:*** We used following types of purposive sampling technique for patients and healthcare providers: maximum variation for patients ( based on a) adverse events- absence or presence of adverse events and b) treatment outcome- on treatment for more than one year or with end-of-treatment outcome within six months of date of interview) and homogeneity for healthcare providers (that is they were supposed to have an experience of providing treatment including Imipenem via port a cath to patients for more than two years)

***Point 8:*** Lines 236 – 238, why you included only 5 patients? Were only 5 patients experienced adverse drug events during treatment and treatment outcomes? Do you think that there was saturation by those 5 patients?

***Response 8:*** We wanted to include the maximum variation in patients according to the adverse events and treatment outcome, though it was difficult to interview many patients (due to operational challenges and poor clinical condition of patients). We understand the reviewers concern, however we believe the thematic analysis including the perspectives of patients and healthcare providers provide close to saturation for the line of query.

***Point 9:*** Moreover, please specify the patients´ status during the interview, whether they were on treatment or completed the treatment.

***Response 9:*** Three patients had completed the treatment while two patients were on treatment at the time of interview. The two patients on treatment had completed more than 12 months of treatment. We have included this information in the results section.

***Point 10:*** Data collection: line 251, were the interview guides translated to local language?

***Response 10:*** Yes the interview guides were translated in local language (Hindi). We have added the same information in the manuscript.

***Point 11:*** Lines 258 and 259, “...the interviews were audio recorded and lasted for on average 28 (range: 8-63) mins. The minimum time taken for the interview was too short (8 mins), were these interviews lasted for few mins ( 8, 9, 10 mins ) complete? OR incomplete? As a qualitative study, it might be difficult to explore what was expected within the short time.

***Response 11:*** We understand reviewer’s concern. We agree it is difficult to explore the findings within short period of time, but the interviewer tried to cover all the lines of queries in the available time period. We continued the interviews till the time patients were comfortable.

***Point 12:*** What data quality assurance methods you have used for the qualitative data? (for example, building trust, small talk before formal interview, member check, debriefing, ….)

***Response 12:*** The identified eligible participants were explained the purpose of the study, study objectives, and process of interviews (including consent for audio-recording) in detail by the interviewer with support of the healthcare provider. If the participants agreed, informed consent was taken from the participants. During the time of interview, the interviewer repeated about the data confidentiality and safety procedures, and answered all the queries of the participant before the start of the interview. At the end of each interview, the interviewer shared the summary of findings for participant validation. Similar texts have been added in the manuscript.

***Point 13:*** Data analysis: lines 276 and 277, “The interviewer prepared the transcripts within two days of conducting the interview” why the interviewer waited for two days? Why not did daily transcription? Moreover, please describe the whole analysis method of qualitative data (codes, categories, and themes).

***Response 13:*** Two interviews were scheduled in one day. The interviews were audio-recorded and transcripts were prepared within two days. Most of the transcripts were prepared on same day, but the transcripts of two of the interviews were prepared on second day, due to operational challenges. We utilised thematic network analysis as our framework for analysis, as described by Attride-Stirling. The thematic network facilitated the structuring of textual data and depiction of main themes in one nexus. The codes were identified, which were grouped under categories or sub-themes and thereafter as themes. Similar information is added in the manuscript under data analysis section.

***Results***

***Point 14:*** Line 349, there should be categories/ sub-themes between codes and themes. The same is true in the method section.

***Response 14:*** The overarching theme of the qualitative analysis was: Challenges in delivering Imipenem via port-a-cath device in ambulatory care. The over-arching theme consisted of four sub-themes: 1) Patient related challenges, 2) Imipenem related challenges, 3) Port-a-cath related challenges and 4) Home based care related. A total of 26 codes were identified under these sub-themes. We have added this description in the results section of the manuscript.

***Point 15:*** For qualitative part/ challenges: you mentioned repeatedly “The healthcare providers, the patients, or the healthcare providers and patients reported/ felt/ expressed/ mentioned … “ Do you mean all, a majority, most, or some? Please specify for the sentences before the quotes. It is obvious that a single person reported what was mentioned in the quote.

***Response 15:*** Yes the challenges identified were mentioned by most of the participants. As we are cognizant of the word count, we have only picked up one quote per identified code.

***Discussion***

***Point 16:*** Line 480, “ … treatment outcomes in the current cohort was poor compared to Nix-TB trial with all oral shorter regimen…”, please explain the possible reasons or justify after comparing with similar study.

***Response 16:*** we agree that treatment outcomes are poor compared to Nix-TB trial , we would like to restate that while we would like an all oral drug regimen for our patients , availability of drug eg. Pretomanid which is currently not available in our country and history of prior exposure to core drugs like Linezolid for more than two years and severe adverse events to core drugs would result in very few effective oral drugs making the treatment ineffective. injectable drugs would have to be included in the regimen to make it effective. Our study has patients who were started with Imipenem based regimen since an ‘ All Oral Drug ‘ regimen was not possible . We have mentioned in our discussion lines 564-566.

***Point 17:*** As a mixed study, qualitative study findings were not discussed. Need to discuss the qualitative study findings too.

***Response 17:*** Thank you for the comment. However, we feel the findings of the qualitative component are context specific and might not be relevant to compare with findings in other settings. Also, as this was the first time the changes with ambulatory port-a-cath were explored, we did not have similar literature. However, we have used the insights from the qualitative component extensively in discussing the implications of the study findings. It would be helpful if the reviewer has any specific comments to discuss.

***END***
